# Depression among HIV positive pregnant women in Zimbabwe: a primary health care based cross-sectional study

**DOI:** 10.1186/s12884-019-2193-y

**Published:** 2019-01-31

**Authors:** Eugenia Nyamukoho, Walter Mangezi, Bazondlile Marimbe, Ruth Verhey, Dixon Chibanda

**Affiliations:** Department of Psychiatry & Research Support Centre, University of Zimbabwe, Harare, Zimbabwe

**Keywords:** Depression, HIV positive, Pregnant women

## Abstract

**Background:**

Depression is a common psychiatric disorder that is highly prevalent among people living with HIV (PLWH). Depression is linked to poor adherence to anti-retroviral medication while in the peri-natal period may affect birth outcomes. Intimate partner violence (IPV) has been linked to depression. Little is known about the factors associated with depression in HIV positive pregnant women in Zimbabwe.

**Methods:**

We carried out a cross-sectional study in 4 busy primary care clinics offering antenatal services during the months of June through to September in 2016. Simple random sampling was used to screen HIV positive pregnant women while they waited to be attended to at each clinic. Eligible women who gave written informed consent were screened using a locally validated screening tool-the Edinburgh Postnatal Depression Scale (EPDS).

**Results:**

A total of 198(85%) participants were recruited out of 234 that were approached. The mean age of participants was 26.6(SD 4.5), of these, 176 (88.9%) had secondary education or more. A total of 78 (39.4%) (95% CI 32.5–46.3) met criteria for antenatal depression according to the local version of the EPDS. Factors associated with antenatal depression after multivariate analysis were intimate partner violence (IPV) [OR 3.2 (95% CI 1.5–6.7)] and previous history of depression OR 4.1 (95% CI 2.0–8.0)].

**Conclusion:**

The prevalence of antenatal depression among HIV positive pregnant women in primary care clinics is high. Factors associated with antenatal depression in pregnant HIV positive women are IPV and previous history of depression. There is need for routine screening for depression during the antenatal period and interventions targeting depression in this population should include components to address IPV.

## Background

Sub Saharan Africa (SSA) is the epicentre of the HIV/AIDS pandemic with over 60% of all global cases [1]. In SSA, women constitute 59% of those living with HIV [2] with women of child-bearing age most affected [3].

Depression is highly prevalent in people living with HIV (PLWH), particularly in SSA [2]. Untreated, depression may enhance HIV disease progression through both biological and social mechanisms [4, 5]. For instance adherence to anti-retroviral treatment (ART) among PLWH with depression has consistently been found to be poor [5–7] with a recent cohort study showing depression to have a dose response effect on mortality among PLWH [8]. HIV positive women are particularly vulnerable to depression [9, 10]. In pregnancy rates of depression ranging from 12 to 30% have been reported through a number of systematic reviews [11–13] and regional studies [9, 14–17].

In Zimbabwe, little is known about antenatal depression [18]. However, earlier studies on postnatal depression (PND) have shown rates ranging from 16 to 30% [19, 20] with similar rates reported in the region through a systematic review [21]. There is evidence suggesting that PND often first manifests in the antenatal period [20] therefore identifying and treating pregnant women with depression may lead to better health outcomes for both mother and infant [22].

Evidence based interventions for PLWH affected by depression are known to improve HIV disease outcomes [23]. Most of these interventions based on task-shifting have shown promising results in SSA, including Zimbabwe [24–29].

The aim of this study was to determine the prevalence and factors associated with depression in the antenatal period as part of a policy brief aimed at justifying the introduction of a care program for depression during the antenatal period for HIV positive pregnant women. Furthermore, based on earlier work highlighting intimate partner violence (IPV) as a factor associated with depression in women, [19, 25] we sought to establish the magnitude of IPV in pregnant HIV positive women.

## Methods

### Study design

We conducted a cross-sectional survey of HIV positive pregnant women attending the Chitungwiza City Council antenatal clinics outside of Harare, the capital city of Zimbabwe.

### Study area

Chitungwiza is a high density suburb situated about 30 km south of Harare. It has four city council clinics which provide services to an estimated 1.2 million people. Each clinic offers general medical and surgical care, as well as maternity services provided by nurses and visiting medical doctors. All 4 clinics also offer provider initiated testing and counselling for HIV, including initiation of anti-retroviral treatment (ARV) and follow up of PLWH. Cases that cannot be managed at clinic level are referred to Chitungwiza Central Hospital which is run by specialised staff.

The maternity services offered in Chitungwiza commence during the antenatal period when pregnant women register for delivery. During registration all women are pre-test counselled, tested for HIV and are post-test counselled. Those found to be HIV positive are counselled further and initiated on ARV on the same day (Option B+).

### Participants

All pregnant HIV positive women in the first trimester booked at Chitungwiza city council antenatal clinics were eligible for inclusion in the study.

Those who were HIV negative, or had an unconfirmed HIV positive result, showed psychotic symptoms, an intellectual disability or could not give informed consent were excluded from the study.

### Sampling/recruitment

The sampling frame consisted of all antenatal clinic attendees registered as HIV positive utilising the facilities between 14 June 2016 to 14 September 2016. Each day during this period participants were approached based on a computer generated random number allocation. They were initially informed about the study and all those who gave initial verbal consent were further assessed for inclusion criteria which included age > 17 and a confirmed HIV+ test result. Those that met this criteria were then informed in detail of the study and if they still were interested written consent was sought.

### Screening

The Edinburgh Postnatal Depression Scale (EPDS) was used to screen for antenatal depression in all 4 clinics by trained research assistants. The EPDS has been validated in Zimbabwe in the postnatal period and found to have good psychometric properties with sensitivity and specificity of 88 and 87% respectively. It has a Chronbach’s alpha of 0.87 at cut-off of 11/12 [30]. The EPDS is a widely used screening tool in sub-Saharan Africa with most settings using a cut-off score of between 12 and 15 [21]. It consists of 10 questions which ask about events in the past seven days. The last question assesses thoughts of self-harm and women who score anything other than 0 on this question need further assessment. Each response is scored from 0 to 3, with responses to questions 3, 5 to 10 reverse scored. The total score ranges between 0 and 30. Common cut-off scores for the EPDS range between 9 and 13 [9, 21, 30, 31].

Part of the study involved training research assistants (RA) in the administration of the EPDS and the socio-demographic questionnaire. This activity was carried out by the first author (EN) who also run a pilot before formal recruitment of study participants. The pilot included checking completeness of collected data, and crosschecking information with the antenatal register at each clinic for gestational age and date of HIV testing.

### Socio-demographic

A questionnaire on socio-demographic characteristics was interviewer administered. It included questions about; when participant found out about their HIV positive status, history of intimate partner violence, history of childhood sexual abuse, previous episode of depression, and type of anti-retroviral treatment (ARVs) that participants were taking including duration in months of taking ARVs. We also captured significant life events in the past 3 months and the nature of the events. Previous history of depression was determined through a series of questions derived from the Diagnostic Statistical Manual (DSM) [32] which we had piloted in a previous study [19]. Intimate partner violence (IPV) was defined as physical violence, forced sexual intercourse, committed by a partner in an intimate relationship [33]. All other socio-demographic information used for this study was based on previous local studies in similar populations [18–20]. A two week period was used to pre-test and pilot tools by the first author EN.

### Analysis

The Statistical package for Social Scientist (SPSS) version 16.0 was utilised for data analysis. Continuous variable results were presented as means ± SEM. Categorical variables were expressed as percentages and frequencies, and compared using the Chi-square test to compute *p*-values. Binary logistic regression was used to find significant predictors of depression and also to estimate odds ratios and 95% confidence intervals for the risk factors. All statistical significance was evaluated at *p* < 0.05 (2 sided). A logistic regression analysis was conducted to predict depression among HIV positive mothers of 198 participants using the variables in the table (Table 1)*.* A test of the full model against a constant only model was carried out to determine significance and to indicate that the predictors as a set reliably distinguished between those who were depressed and non-depressed. Nagelkerke’s R^2^ was measured to determine strength of relationship.

**Table 1:** Characteristics of study participants by depression

	Overall	Not Depressed	Depressed
	*N* (%)	*N* (%)	*N* (%)
Age			
< 24	42 (21.1%)	24 (20.0%)	18 (23.1%)
25–34	115 (58.1%)	71 (59.2%)	44 (56.4%)
> 35	41 (20.7%)	25 (20.8%)	16 (20.5%)
Education			
Primary	22 (11.1%)	11 (9.2%)	11 (14.1%)
Secondary	169 (85.4%)	106 (88.3%)	63 (80.8%)
Tertiary	7 (3.5%)	3 (2.5%)	4 (5.1%)
Marital status			
Divorced	3 (1.5%)	0 (0.0%)	3 (3.8%)
Married	188 (94.9%)	117 (97.5%)	71 (91.0%)
Never married	3 (1.5%)	1 (0.8%)	2 (2.6%)
Separated	3 (1.5%)	1 (0.8%)	2 (2.6%)
Widowed	1 (0.5%)	1 (0.8%)	0 (0.0%)
Employment status			
Formally employed	17 (8.6%)	11 (9.2%)	6 (7.7%)
Informally employed	55 (27.8%)	33 (27.5%)	22 (28.2%)
Unemployed	126 (63.6%	76 (63.3%)	50 (64.1%)
Know HIV status			
Before current pregnancy	113 (57.1%)	68 (56.7%)	45 (57.7%)
During current pregnancy	85 (42.9%)	52 (43.3%)	33 (42.3%)
Intimate partner violence			
No	153 (77.3%)	104 (86.7%)	49 (62.8%)
Yes	45 (22.7%)	16 (13.3%)	29 (37.2%)
Childhood sexual abuse			
No	169 (85.4%)	108 (90.0%)	61 (78.2%)
Yes	29 (14.6%)	12 (10.0%)	17 (21.8%)
Social support			
No	17 (8.6%)	13 (10.8%)	4 (5.1%)
Yes	181 (91.4%)	107 (89.2%)	74 (94.9%)
History of depression			
No	142 (71.7%)	101 (84.2%)	41 (52.6%)
Yes	56 (28.3%)	19 (15.8%)	37 (47.4%)
On ARVs			
No	4 (2.0%)	2 (1.7%)	2 (2.6%)
Yes	194 (98.0%)	118 (98.3%)	76 (97.4%)
Children			
No	39 (19.7%)	24 (20.0%)	15 (19.2%)
Yes	159 (80.3%)	96 (80.0%)	63 (80.8%)
Ever had a miscarriage			
No	167 (84.3%)	102 (85.0%)	65 (83.3%)
Yes	31 (15.7%)	18 (15.0%)	13 (16.7%)
Chronic condition			
No	189 (95.5%)	116 (96.7%)	73 (93.6%)
Yes	9 (4.5%)	4 (3.3%)	5 (6.4%)

Furthermore, during analysis where there was no pattern in missing data on any variables these responses were omitted.

### Ethical consideration

Ethical approval was sought from the Joint Research Ethical Committee (JREC), Chitungwiza City Council Department of Health and the Medical Research Council of Zimbabwe (MRCZ). Written informed consent was obtained from everyone who agreed to participate in the study. Interviews were held in private consultation rooms for privacy. To maintain confidentiality, study participants were identified using study participant numbers.

Only a few people had access to the study material of study participants, that is: the first author, last author and 2 research assistants involved in entering the data into a desktop computer.

Research material was kept locked in a safe in the department of Psychiatry. Those who were found to be severely depressed or suicidal were referred to a visiting Psychiatrist at Chitungwiza Central Hospital.

## Results

A total of 234 women were randomly approached with 198 (85%) of them giving written informed consent to participate in the study. From the 198 women, 78 (39.4%), 95% CI 32.5–46.3 met depression criteria according to the EPDS using the local cut-off score of 12 > [22]. Most of the participants (58%) were aged between 25 and 34 years with the mean age being 26.6 (SD4.5). There was no statistically significant association between age and antenatal depression. One hundred and ninety-four (98%) of all participants were taking anti-retroviral medication (ARVs). A total of 45 (22.7%) participants reported intimate partner violence (IPV) (Table 1).

Univariate analysis showed significant odds ratios (OR) for IPV [OR 3.8 (95% CI 1.97–3.8)], childhood sexual abuse (CSA) [OR2.5 (95% CI 1.1–5.5)] and previous history of depression [OR4.8. 95% CI 2.5–9.3)], however, on multi-variate analysis IPV [OR3.2 95% CI 1.5–6.7)] and history of depression [OR 4.1 95% CI 2.0–8.0)] were the only two variables that were statistically significant Table 2.

**Table 2:** Factors associated with depression by EPDS

		Overall	Not Depressed	Depressed	*P*-value	OR (95% CI)
		*N* (%)	*N* (%)	*N* (%)		
Age	< 24	42 (21.1%)	24 (20.0%)	18 (23.1%)	0.87	
	25–34	115 (58.1%)	71 (59.2%)	44 (56.4%)		
	> 35	41 (20.7%)	25 (20.8%)	16 (20.5%)		
Education	Primary	22 (11.1%)	11 (9.2%)	11 (14.1%)	0.32	
	Secondary	169 (85.4%)	106 (88.3%)	63 (80.8%)		
	Tertiary	7 (3.5%)	3 (2.5%)	4 (5.1%)		
Marital status	Divorced	3 (1.5%)	0 (0.0%)	3 (3.8%)	0.11	
	Married	188 (94.9%)	117 (97.5%)	71 (91.0%)		
	Never married	3 (1.5%)	1 (0.8%)	2 (2.6%)		
	Separated	3 (1.5%)	1 (0.8%)	2 (2.6%)		
	Widowed	1 (0.5%)	1 (0.8%)	0 (0.0%)		
Employment status	Formally employed	17 (8.6%)	11 (9.2%)	6 (7.7%)	0.39	
	Informally employed	55 (27.8%)	33 (27.5%)	22 (28.2%)		
	Unemployed	126 (63.6%	76 (63.3%)	50 (64.1%)		
Know HIV status	Before pregnancy	113 (57.1%)	68 (56.7%)	45 (57.7%)	0.88	
	During pregnancy	85 (42.9%)	52 (43.3%)	33 (42.3%)		
IPV	No	153 (77.3%)	104 (86.7%)	49 (62.8%)		
	Yes	45 (22.7%)	16 (13.3%)	29 (37.2%)	0.02	3.8 (1.9–7.7)
CSA	No	169 (85.4%)	108 (90.0%)	61 (78.2%)		
	Yes	29 (14.6%)	12 (10.0%)	17 (21.8%)	0.02	2.5 (1.1–5.6)
Social support	No	17 (8.6%)	13 (10.8%)	4 (5.1%)		
	Yes	181 (91.4%)	107 (89.2%)	74 (94.9%)	0.02	
History of depression	No	142 (71.7%)	101 (84.2%)	41 (52.6%)		
	Yes	56 (28.3%)	19 (15.8%)	37 (47.4%)	0.00	4.8 (2.5–9.3)
On ARVs	No	4 (2.0%)	2 (1.7%)	2 (2.6%)		
	Yes	194 (98.0%)	118 (98.3%)	76 (97.4%)	0.64	
Children	No	39 (19.7%)	24 (20.0%)	15 (19.2%)		
	Yes	159 (80.3%)	96 (80.0%)	63 (80.8%)	0.1	
Ever had miscarriage	No	167 (84.3%)	102 (85.0%)	65 (83.3%)		
	Yes	31 (15.7%)	18 (15.0%)	13 (16.7%)	0.84	
Chronic condition	No	189 (95.5%)	116 (96.7%)	73 (93.6%)	0.31	
	Yes	9 (4.5%)	4 (3.3%)	5 (6.4%)		

## Discussion

This study carried out in 4 busy primary health care facilities in Zimbabwe showed high rates for antenatal depression (39.4%) as measured by the EPDS with IPV and previous history of depression being associated with the condition. These findings are consistent with an earlier systematic review showing that depression is common in pregnant HIV positive women and predicts non-adherence to ART treatment [22]. Our earlier work using the Shona Symptom Questionnaire (SSQ-14) [34] in Zimbabwe revealed a prevalence of 19% among HIV negative women [20]. We are not aware of other studies that have looked at the antenatal period among HIV positive pregnant women in the country. However, the prevalence of PND in a population of women attending the 6 -week post-natal clinic visit was found to be 17% using the SSQ-14 [18] and 33% [19] using the validated EPDS [30] but both these studies had mixed populations consisting of HIV positive and negative women with varying socio-demographic characteristics. Pregnant women are at an increased risk of new onset depression [3, 17] and our findings reflect those of earlier studies carried out in the region. In Uganda a rate of 39% for depression among pregnant women was found using the Hopkins Symptoms Checklist as a screening tool [17], while a prevalence of 48.7% among HIV infected women in rural South Africa was reported using the EPDS at a cut-off point of 13 [35]. In Ethiopia, using the EPDS the prevalence of antenatal depression was found to be 24.94% [16], while in Zambia, 85% of HIV positive pregnant women met criteria for depression [36]. Rates described above are largely based on screening tools which invariably give varying rates based on psychometric properties of the tools. A study from South Africa found rates of 47% for depression using clinical examination based on a gold standard- The Diagnostic Statistical Manual (DSMIV) [15], suggesting that rates are generally high in the region. Identifying women at risk of antenatal depression early during pregnancy is therefore important in order to facilitate early referral to evidence based care programs [22]. Depression during pregnancy can be associated with obstetric complications and is known to increase risk of poor infant outcomes such as delayed developmental milestones [17, 37] therefore early detection of affected women is critical. Our findings indicate that Intimate partner violence (IPV) is highly prevalent among pregnant women. This reflects the growing concern in low and middle income countries (LMIC) showing that an estimated 60% of women in Africa are affected by IPV which is closely linked to depression [38]. A recent systematic review from LMIC on IPV and perinatal mental disorders during pregnancy revealed that participants who had experienced IPV had a 1.69–3.76 and 1.46–7.04 higher odds of antenatal and postnatal depression compared to those who had no IPV [39]. The prevalence of physical IPV has been found to be as high as 35% while sexual IPV and psychological IPV were as high as 40 and 65% respectively [39]. In Zimbabwe, a recent cluster randomised controlled trial of a brief psychological intervention delivered by lay health workers for common mental disorders (CMD) which include depression recorded IPV (physical violence) in 70.1% of those recruited [25]. Similar findings have been reported in India [40]. There is need to develop interventions that take into consideration IPV in women with depression.

Recently, collaborative care interventions have been found to be effective for treating depressed women in non-mental health settings [41]. Furthermore there is growing evidence supporting the use of non-professionals who are trained and supervised to treat depression at primary health care level [42]. Both brief and long form screening tools for depression have been found to be effective in LMIC and can be used by lay health workers (LHWs) as effective ways of identifying women at risk [43], with evidence suggesting that in illiterate populations visual screens could be an effective alternative for identifying those in need of care for depression [44].

In Zimbabwe, the scaling up of an evidence based collaborative care model for depression-the Friendship bench [45] has resulted in improved access to care for CMD at PHC level using trained LHWs who provide a problem solving therapy (PST) treatment [46] for those identified with CMD. A locally validated tool the Shona Symptom Questionnaire-14 [47] is the main screening instrument. While the PST approach provided by the elderly LHWs has been effective [25], there has been no structured focus on women who report IPV during sessions on the Friendship Bench, particularly pregnant women. Furthermore, despite earlier studies showing effectiveness of group PST for women during the postnatal period [24], the health authorities have been slow to integrate such evidence based care packages in antenatal care services due to lack of specific evidence related to the antenatal period. The evidence provided through this study will go towards the compilation of a policy brief aimed at justifying the introduction of depression treatment during the antenatal period.

### Limitations

Although this study shows a high prevalence of depression among women living with HIV attending the antenatal care clinics in Zimbabwe, the use of the EPDS while convenient does have limitations: The EPDS does not provide a definitive diagnosis of depression as defined by either the DSM [32] or the International Classification of Disease version 10 (ICD-10) [48] but instead informs of the probability of an individual having depression. A clinical interview to confirm the diagnosis would provide a more accurate rate of depression. Secondly, although the EPDS is recommended for antenatal screening [49] it has not been validated in the antenatal period in Zimbabwe. In addition the self-reported symptoms of previous episode of depression were not validated by checking the participants medical history which introduces recall bias. Similarly, IPV did not include psychological IPV as defined by Krug [50].

## Conclusion

Despite these limitations our study reflects findings similar to those from the region and beyond suggesting that depression in the antenatal period among HIV infected women is common [22, 35, 51]. It also highlights the need to focus on IPV which is a growing global problem. Using non-professionals who are trained to screen and identify those with probable depression during pregnancy and provide evidence based care management could considerably reduce the treatment gap for depression in pregnancy. A recent systematic review on IPV suggests that a problem solving therapy approach similar to that being offered by the Friendship Bench could address most issues linked to IPV [52]. Furthermore, strengthening existing legislation aimed at preventing IPV should be prioritised by governments.

## Abbreviations

CMD: Common mental disorders
EPDS: Edinburgh Post-natal depression scale
IPV: Intimate partner violence
LHW: Lay health workers
PLWH: People living with HIV
SSQ-14: Shona symptom questionnaire-14
